# Two Dyakonov–Voigt surface waves guided by a biaxial–isotropic dielectric interface

**DOI:** 10.1038/s41598-020-69727-z

**Published:** 2020-07-30

**Authors:** Chenzhang Zhou, Tom G. Mackay, Akhlesh Lakhtakia

**Affiliations:** 1 NanoMM, Nanoengineered Metamaterials Group, Department of Engineering Science and Mechanics, Pennsylvania State University, University Park, PA 16802–6812 USA; 2 School of Mathematics and Maxwell Institute for Mathematical Sciences, University of Edinburgh, Edinburgh, EH9 3FD UK

**Keywords:** Optical materials and structures, Other photonics

## Abstract

Electromagnetic surface waves guided by the planar interface of an orthorhombic dielectric material and an isotropic dielectric material were analyzed theoretically and numerically. Both naturally occurring minerals (crocoite, tellurite, and cerussite) and engineered materials were considered as the orthorhombic partnering material. In addition to conventional Dyakonov surface waves, the analysis revealed that as many as two Dyakonov–Voigt surface waves can propagate in each quadrant of the interface plane, depending upon the birefringence of the orthorhombic partnering material. The coexistence of two Dyakonov–Voigt surface waves marks a fundamental departure from the corresponding case involving the planar interface of a uniaxial dielectric material and an isotropic dielectric material for which only one Dyakonov–Voigt surface wave is possible. The two Dyakonov–Voigt surface waves propagate in different directions in each quadrant of the interface plane, with different relative phase speeds and different penetration depths. Furthermore, the localization characteristics of the two Dyakonov–Voigt surface waves at the planar interface are quite different: the Dyakonov–Voigt surface wave with the higher relative phase speed is much less tightly localized at the interface in the isotropic dielectric partnering material.

## Introduction

Any electromagnetic surface wave is guided by the planar interface of two dissimilar partnering materials^1,2^. The characteristics of a surface wave are determined by the constitutive properties of the two partnering materials. If the constitutive properties of both partnering materials are independent of direction (i.e., if both partnering materials are isotropic), then the properties of the surface waves will also be independent of the propagation direction; on the other hand, if the constitutive properties of at least one of the partnering materials depend on direction (i.e., if at least one of the partnering materials is anisotropic), then the properties of the surface waves will also depend on the propagation direction. For example, surface-plasmon-polariton waves are guided by the planar interface of a plasmonic material and a dielectric material (such as air or quartz)^3,4^, the real part of the permittivity dyadic of the plasmonic material being negative definite and the real part of the permittivity dyadic of the dielectric material being positive definite^5^. Likewise, Dyakonov surface waves are guided by the planar interface of two dielectric materials with at least one being anisotropic^6–9^. Unlike for surface-plasmon-polariton waves, dissipation in both partnering materials for Dyakonov surface waves can be negligibly small, which makes these surface waves attractive candidates for applications involving long-range optical communications.

In addition to Dyakonov surface waves, it was recently discovered that the planar interface of a uniaxial dielectric material and an isotropic dielectric material can guide the propagation of Dyakonov–Voigt surface waves^10,11^. As compared to conventional surface waves such as surface-plasmon-polariton waves and Dyakonov surface waves, Dyakonov–Voigt surface waves have quite different localization characteristics. That is, the fields of conventional surface waves decay exponentially with distance from the interface whereas the decay of fields of a Dyakonov–Voigt surface wave is specified by the product of an exponential function and a linear function of distance from the interface in the anisotropic partnering material. Furthermore, for the planar interface of a uniaxial dielectric material and an isotropic dielectric material, Dyakonov–Voigt surface waves propagate in only one direction for each quadrant of the interface plane; in contrast, conventional surface waves such as surface-plasmon-polariton waves and Dyakonov surface waves propagate in a continuous range of directions in the interface plane. Dyakonov–Voigt surface waves arise as manifestations of exceptional points^12^ of a $4\times 4$ matrix that governs propagation in the anisotropic partnering material^13^, that matrix being non-diagonalizable at these exceptional points.

Dyakonov–Voigt surface waves have some similarities to Voigt plane waves that propagate in certain directions in certain unbounded anisotropic^14,15^ and bianisotropic^16,17^ materials. Notably, Voigt waves arise as manifestations of exceptional points of a propagation matrix, when the matrix is non-diagonalizable^18^. And the fields of Voigt waves decay as the product of a linear function and an exponential function of propagation distance. However, the material supporting Voigt-wave propagation must be dissipative^19–21^ (or active^22^), unlike the case for Dyakonov–Voigt surface waves.

In the following sections, Dyakonov–Voigt surface-wave propagation is explored for the planar interface of a biaxial dielectric material, labeled $\mathcal{A}$, and an isotropic dielectric material, labeled $\mathcal{B}$. Both partnering materials $\mathcal{A}$ and $\mathcal{B}$ are homogeneous and nondissipative. Dyakonov surface waves for interfaces involving biaxial materials have been studied previously^23–25^ but Dyakonov–Voigt surface waves have not. Our analysis reveals that one or two Dyakonov–Voigt surface waves can be guided for each quadrant of the interface plane, depending upon the birefringence of partnering material $\mathcal{A}$. The manifestation of *two* Dyakonov–Voigt surface waves (rather than just *one*^13^) marks a fundamental departure from the case involving the planar interface of a uniaxial dielectric material and an isotropic dielectric material^10,11^.

As regards the notation adopted, the permittivity and permeability of free space are written as $\varepsilon _{\scriptscriptstyle 0}$ and $\mu _{\scriptscriptstyle 0}$, respectively. The free-space wavenumber, wavelength, and impedance are written as $k_{\scriptscriptstyle 0}= \omega \sqrt{\varepsilon _{\scriptscriptstyle 0}\mu _{\scriptscriptstyle 0}}$, $\lambda _{\scriptscriptstyle 0}= 2 \pi / k_{\scriptscriptstyle 0}$, and $\eta _{\scriptscriptstyle 0}= \sqrt{\mu _{\scriptscriptstyle 0}/\varepsilon _{\scriptscriptstyle 0}}$, respectively, with $\omega$ being the angular frequency. Single underlining signifies a 3-vector and $\left\{ \hat{\underline{u}}_x, \hat{\underline{u}}_y, \hat{\underline{u}}_z\right\}$ is the triad of unit vectors aligned with the Cartesian axes. Matrixes and column vectors are enclosed by square brackets. The superscript ${}^T$ denotes the transpose. The operators $\text{ Re } \left\{ {{^\cdot }}\right\}$ and $\text{ Im } \left\{ {{^\cdot }}\right\}$ deliver the real and imaginary parts, respectively, of complex-valued quantities; the complex conjugate is denoted by an asterisk; and dependence on time *t* is achieved implicitly through $\exp (-i\omega t)$ with $i = \sqrt{-1}$.

## Canonical boundary-value problem

### $4\times 4$ matrix ordinary-differential-equation formalism

**Figure 1:**
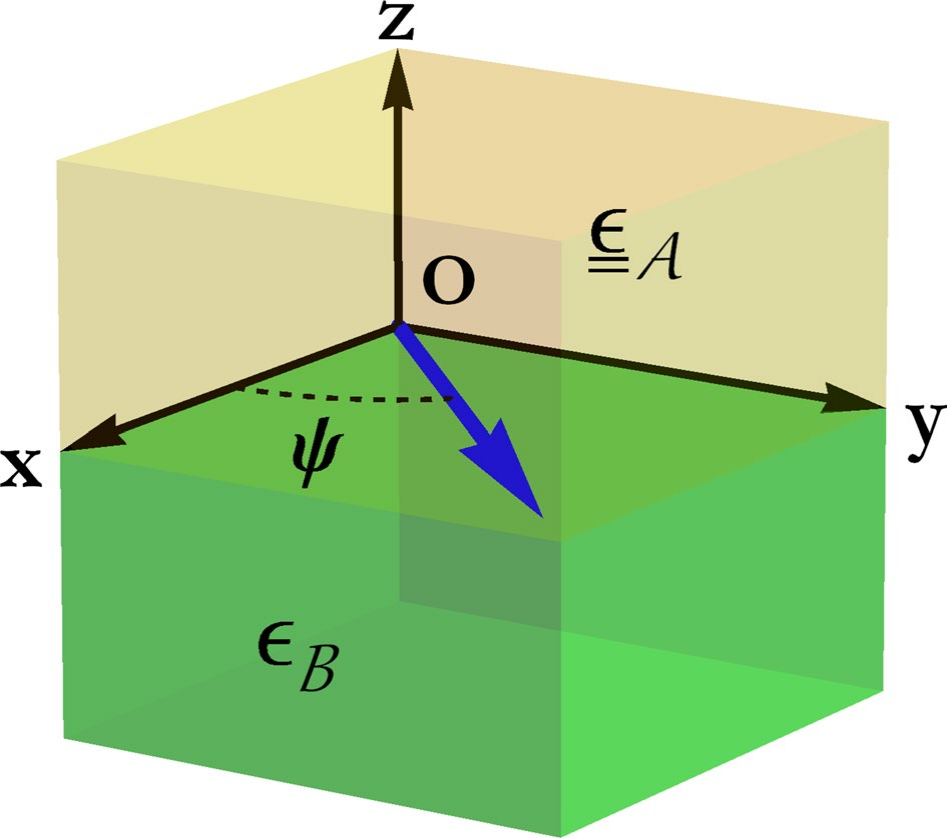
A schematic illustration of the canonical boundary-value problem for Dyakonov–Voigt (and Dyakonov) surface waves that propagate parallel to the interface plane $z=0$ at the angle $\psi$ relative to the *x* axis.

In the canonical boundary-value problem for Dyakonov–Voigt (and Dyakonov) surface-wave propagation, material $\mathcal{A}$ fills the half-space $z>0$ and material $\mathcal{B}$ the half-space $z<0$, as illustrated schematically in Fig. 1.

Dissipation is assumed to be negligibly small in both materials. Material $\mathcal{A}$ is a biaxial dielectric material specified by the relative permittivity dyadic^26,27^

1 $$\begin{aligned} \underline{\underline{\varepsilon }}_{\mathcal{A}} = \varepsilon _{\mathcal{A}a}\,\hat{\underline{u}}_z\,\hat{\underline{u}}_z+ \varepsilon _{\mathcal{A}b}\, \hat{\underline{u}}_x\,\hat{\underline{u}}_x+ \varepsilon _{\mathcal{A}c}\, \hat{\underline{u}}_y\,\hat{\underline{u}}_y, \end{aligned}$$

with $\varepsilon _{\mathcal{A}a}$, $\varepsilon _{\mathcal{A}b}$, and $\varepsilon _{\mathcal{A}c}$ being the principal relative permittivity scalars. Material $\mathcal{A}$ belongs to the orthorhombic crystallographic class^28^. In the arena of crystal optics^29^, $\varepsilon _{\mathcal{A}a}>1$, $\varepsilon _{\mathcal{A}b}>1$, and $\varepsilon _{\mathcal{A}c}>1$. The birefringence of material $\mathcal{A}$ is the excess of the square root of the largest principal relative permittivity scalar over the square root of the smallest principal relative permittivity scalar^25^. We take material $\mathcal{B}$ as an isotropic dielectric material specified by the relative permittivity $\varepsilon _{\mathcal{B}}>1$.

The electromagnetic field phasors for surface-wave propagation are expressed everywhere as^2^

2 $$\begin{aligned} \left. \begin{array}{l} \underline{E} (\underline{r})= \left[ e_x(z)\hat{\underline{u}}_x+ e_y(z)\hat{\underline{u}}_y+e_z(z)\hat{\underline{u}}_z\right] \, \exp \left[ i q \left( x \cos \psi + y \sin \psi \right) \right] \\ \underline{H} (\underline{r})= \left[ h_x(z)\hat{\underline{u}}_x+ h_y(z)\hat{\underline{u}}_y+h_z(z)\hat{\underline{u}}_z\right] \, \exp \left[ i q \left( x \cos \psi + y \sin \psi \right) \right] \end{array}\right\} , \,\,\, -\infty< z < + \infty , \end{aligned}$$

with *q* being the surface wavenumber. The direction of propagation in the *xy* plane, relative to the *x* axis, is prescribed by the angle $\psi \in \left[ 0,2\pi \right)$. Substitution of the phasor representations (2) in the source-free Maxwell curl equations yields the $4\times 4$ matrix ordinary differential equations^30,31^

3 $$\begin{aligned} \frac{d}{dz}[\underline{f}(z)]= \left\{ \begin{array}{l} i [\underline{\underline{P}}_{\mathcal{A}}]{{^\cdot }}[\underline{f}(z)], \qquad z>0 \\ i [\underline{\underline{P}}_{\mathcal{B}}]{{^\cdot }}[\underline{f}(z)], \qquad z<0 \end{array} , \right. \end{aligned}$$

wherein the column 4-vector

4 $$\begin{aligned}{}[\underline{f}(z)]= [\begin{array}{c} e_ x(z), \quad e_y(z),\quad h_x(z),\quad h_y(z) \end{array} ]^T, \end{aligned}$$

and the $4\times 4$ propagation matrixes $[\underline{\underline{P}}_{\mathcal{A}}]$ and $[\underline{\underline{P}}_{\mathcal{B}}]$ depend on $\underline{\underline{\varepsilon }}_{\mathcal{A}}$ and $\varepsilon _{\mathcal{B}}$, respectively. The *x*-directed and *y*-directed components of the phasors are algebraically connected to their *z*-directed components^27,31^.

### Fields in material $\mathcal{A}$

The $4\times 4$ propagation matrix

5 $$\begin{aligned}{}[\underline{\underline{P}}_{\mathcal{A}}]= \left[ \begin{array}{cccc} 0&{}0&{} \displaystyle {\frac{\tau }{ \omega \varepsilon _{\scriptscriptstyle 0}\varepsilon _{\mathcal{A}a}}} &{} \displaystyle { \frac{k_{\scriptscriptstyle 0}^2 \varepsilon _{\mathcal{A}a}- \nu _c}{\omega \varepsilon _{\scriptscriptstyle 0}\varepsilon _{\mathcal{A}a}} } \\ 0&{}0&{} \displaystyle {\frac{\nu _s -k_{\scriptscriptstyle 0}^2 \varepsilon _{\mathcal{A}a}}{\omega \varepsilon _{\scriptscriptstyle 0}\varepsilon _{\mathcal{A}a}}}&{} \displaystyle {- \frac{\tau }{ \omega \varepsilon _{\scriptscriptstyle 0}\varepsilon _{\mathcal{A}a}}} \\ \displaystyle {-\frac{\tau }{\omega \mu _{\scriptscriptstyle 0}}} &{} \displaystyle {\frac{\nu _c -k_{\scriptscriptstyle 0}^2 \varepsilon _{\mathcal{A}c}}{\omega \mu _{\scriptscriptstyle 0}}} &{}0&{}0 \\ \displaystyle {\frac{k_{\scriptscriptstyle 0}^2 \varepsilon _{\mathcal{A}b}- \nu _s }{\omega \mu _{\scriptscriptstyle 0}}} &{} \displaystyle {\frac{\tau }{\omega \mu _{\scriptscriptstyle 0}}}&{}0&{}0 \end{array} \right] , \end{aligned}$$

contains the scalar quantities

6 $$\begin{aligned} \left. \begin{array}{l} \nu _c = q^2 \cos ^2 \psi \\ \nu _s = q^2 \sin ^2 \psi \\ \tau = q^2 \cos \psi \sin \psi \end{array} \right\} \end{aligned}$$

that depend on the propagation characteristics of the surface wave. The *z*-directed components of the field phasors can be determined as follows:

7 $$\begin{aligned} \left. \begin{array}{l} e_z(z) = \displaystyle {-\,\frac{q \left[ h_ y(z) \cos \psi - h_ x(z) \sin \psi \right] }{\omega \varepsilon _{\scriptscriptstyle 0}\varepsilon _{\mathcal{A}a}}}\\ h_ z(z) = \displaystyle {\frac{q \left[ e_ y(z) \cos \psi - e_ x(z) \sin \psi \right] }{\omega \mu _{\scriptscriptstyle 0}}} \end{array} \right\} ,\qquad z > 0. \end{aligned}$$

#### Dyakonov surface wave

The four eigenvalues of $[\underline{\underline{P}}_{\mathcal{A}}]$ can be written as $\pm \alpha _{\mathcal{A}1}$ and $\pm \alpha _{\mathcal{A}2}$. The two with positive imaginary parts are

8 $$\begin{aligned} \left. \begin{array}{l} \alpha _{\mathcal{A}1} = i \sqrt{ A_1 +A_2 q^2+\sqrt{A_3 + A_4 q^2 + A_5 q^4}} \\ \alpha _{\mathcal{A}2} = i \sqrt{ A_1 +A_2 q^2-\sqrt{A_3 + A_4 q^2 + A_5 q^4}} \end{array} \right\} , \end{aligned}$$

wherein the *q*-independent scalar quantities

9 $$\begin{aligned} \left. \begin{array}{l} A_1 =\displaystyle { -k_{\scriptscriptstyle 0}^2\frac{\varepsilon _{\mathcal{A}b}+\varepsilon _{\mathcal{A}c}}{2}} \\ A_2 = \displaystyle {\frac{\varepsilon _{\mathcal{A}a}+\varepsilon _{\mathcal{A}c}+\left( \varepsilon _{\mathcal{A}b}-\varepsilon _{\mathcal{A}c}\right) \cos ^2 \psi }{2 \varepsilon _{\mathcal{A}a}}} \\ A_3= \displaystyle { k_{\scriptscriptstyle 0}^4 \frac{\left( \varepsilon _{\mathcal{A}b}-\varepsilon _{\mathcal{A}c}\right) ^2}{4}} \\ A_4=\displaystyle { -k_{\scriptscriptstyle 0}^2 \frac{\left( \varepsilon _{\mathcal{A}b}-\varepsilon _{\mathcal{A}c}\right) \left[ \varepsilon _{\mathcal{A}b}-\varepsilon _{\mathcal{A}c}+\left( \varepsilon _{\mathcal{A}b}-2 \varepsilon _{\mathcal{A}a}+\varepsilon _{\mathcal{A}c}\right) \cos 2\psi \right] }{4 \varepsilon _{\mathcal{A}a}}} \\ A_5 =\displaystyle { \frac{\left[ \varepsilon _{\mathcal{A}c}- \varepsilon _{\mathcal{A}a}+\left( \varepsilon _{\mathcal{A}b}-\varepsilon _{\mathcal{A}c}\right) \cos ^2 \psi \right] ^2}{4 \varepsilon _{\mathcal{A}a}^2}} \end{array} \right\} . \end{aligned}$$

The eigenvectors of $[\underline{\underline{P}}_{\mathcal{A}}]$ corresponding to the eigenvalues $\alpha _{\mathcal{A}1}$ and $\alpha _{\mathcal{A}2}$ can be stated as follows:

10 $$\begin{aligned} {[\underline{v}_{\mathcal{A}\ell }]}= \left[ \begin{array}{c} \displaystyle {\frac{k_{\scriptscriptstyle 0}^2 \varepsilon _{\mathcal{A}a}- q^2 - \alpha _{\mathcal{A}\ell }^2 }{k_{\scriptscriptstyle 0}\alpha _{\mathcal{A}\ell } \left( \varepsilon _{\mathcal{A}a}- \varepsilon _{\mathcal{A}b}\right) }} \\ \displaystyle {\frac{q^2 \left( \alpha ^2_{\mathcal{A}\ell }+q^2- \varepsilon _{\mathcal{A}a}k_{\scriptscriptstyle 0}^2 \right) \sin \psi \cos \psi }{k_{\scriptscriptstyle 0}\alpha _{\mathcal{A}\ell }\left\{ \varepsilon _{\mathcal{A}a}\left( \varepsilon _{\mathcal{A}c}k_{\scriptscriptstyle 0}^2 -\alpha ^2_{\mathcal{A}\ell } \right) - q^2 \left[ \varepsilon _{\mathcal{A}c}+ \left( \varepsilon _{\mathcal{A}a}- \varepsilon _{\mathcal{A}c}\right) \cos ^2 \psi \right] \right\} }} \\ \displaystyle {\frac{q^2 \eta _{\scriptscriptstyle 0}^{-1}\left( \varepsilon _{\mathcal{A}a}-\varepsilon _{\mathcal{A}c}\right) \sin \psi \cos \psi }{ \varepsilon _{\mathcal{A}a}\left( \varepsilon _{\mathcal{A}c}k_{\scriptscriptstyle 0}^2 -\alpha ^2_{\mathcal{A}\ell } \right) - q^2 \left[ \varepsilon _{\mathcal{A}c}+ \left( \varepsilon _{\mathcal{A}a}- \varepsilon _{\mathcal{A}c}\right) \cos ^2 \psi \right] }} \\ \displaystyle {\eta _{\scriptscriptstyle 0}^{-1}} \end{array} \right] , \qquad \ell \in \left\{ 1, 2 \right\} . \end{aligned}$$

Hence, the general solution of Eq. (3)${}_1$ is given as

11 $$\begin{aligned} {[\underline{f}(z)]} =C_{\mathcal{A}1} {[\underline{v}_{\mathcal{A}1} ]}\exp \left( i \alpha _{\mathcal{A}1} z \right) + C_{\mathcal{A}2}{[\underline{v}_{\mathcal{A}2}]} \exp \left( i \alpha _{\mathcal{A}2} z \right) ,\qquad z > 0, \end{aligned}$$

for fields that decay as $z \rightarrow +\infty$. The complex-valued constants $C_{\mathcal{A}1}$ and $C_{\mathcal{A}2}$ have to be determined by application of appropriate boundary conditions at $z=0$.

#### Dyakonov–Voigt surface wave

For Dyakonov–Voigt surface-wave propagation, $[\underline{\underline{P}}_{\mathcal{A}}]$ exhibits non-semisimple degeneracy because $\alpha _{\mathcal{A}1} = \alpha _{\mathcal{A}2} \equiv \alpha _{\mathcal{A}}$. Then, $[\underline{\underline{P}}_{\mathcal{A}}]$ has only two eigenvalues, namely $\pm \alpha _{\mathcal{A}}$, each with algebraic multiplicity 2 and geometric multiplicity 1^32^. Unlike the case when material $\mathcal{A}$ is uniaxially dielectric^10^, there are two possible values of *q* that give rise to Dyakonov–Voigt surface waves, namely $q^+$ and $q^-$; from Eqs. (8), these are given as

12 $$\begin{aligned} q^\pm = \sigma \displaystyle {\sqrt{\frac{-A_4 \pm \sqrt{A_4^2-4 A_3 A_5}}{2 A_5}}}. \end{aligned}$$

Herein the sign parameter $\sigma = +1$ for $\psi \in \left( 0, \pi /2 \right)$ and $\sigma = -1$ for $\psi \in \left( \pi /2, \pi \right)$. When $q = q^+$ the eigenvalue $\alpha _{\mathcal{A}}$ takes the value $\alpha ^+_{\mathcal{A}}$, and likewise the eigenvalue $\alpha _{\mathcal{A}}$ takes the value $\alpha ^-_{\mathcal{A}}$ when $q=q^-$, with

13 $$\begin{aligned} \alpha ^\pm _{\mathcal{A}} = \displaystyle {i \sigma \sqrt{A_1+A_2 \left( q^\pm \right) ^2 }}. \end{aligned}$$

The square root in Eq. (13) is selected in order to ensure that $\text{ Im } \left\{ \alpha ^\pm _{\mathcal{A}} \right\} > 0$ which is necessary for surface-wave propagation. The propagation directions prescribed by $\psi =\ell \pi /2$, $\ell \in \left\{ {0,1,2,3}\right\}$, are irrelevant to Dyakonov–Voigt surface-wave propagation.

By inspection of Eqs. (12), the inequality $A^2_4-4 A_3 A_5 >0$ must be satisfied in order to achieve $q^\pm \in \mathbb {R}$; this inequality reduces to

14 $$\begin{aligned} \left( \varepsilon _{\mathcal{A}b}-\varepsilon _{\mathcal{A}a}\right) \left( \varepsilon _{\mathcal{A}a}- \varepsilon _{\mathcal{A}c}\right) >0. \end{aligned}$$

Consequently, either $\varepsilon _{\mathcal{A}b}> \varepsilon _{\mathcal{A}a}> \varepsilon _{\mathcal{A}c}$ or $\varepsilon _{\mathcal{A}b}< \varepsilon _{\mathcal{A}a}< \varepsilon _{\mathcal{A}c}$ is required for non-attenuating Dyakonov-Voigt surface waves. Without loss of generality, $\varepsilon _{\mathcal{A}b}> \varepsilon _{\mathcal{A}a}> \varepsilon _{\mathcal{A}c}$ is assumed henceforth.

An eigenvector of the matrix $[\underline{\underline{P}}_{\mathcal{A}}]$ corresponding to the eigenvalue $\alpha ^\pm _{\mathcal{A}}$ can be written as

15 $$\begin{aligned} {[\underline{v}^\pm _{\mathcal{A}}]} = \left[ \begin{array}{c} \displaystyle {\frac{k_{\scriptscriptstyle 0}^2 \varepsilon _{\mathcal{A}a}- \left( \alpha ^\pm _{\mathcal{A}} \right) ^2 - \left( q^\pm \right) ^2}{k_{\scriptscriptstyle 0}\alpha ^\pm _{\mathcal{A}} \left( \varepsilon _{\mathcal{A}a}-\varepsilon _{\mathcal{A}b}\right) }} \\ \displaystyle {\frac{\left( q^\pm \right) ^2 \left[ \left( q^\pm \right) ^2 +{ \left( \alpha ^\pm _{\mathcal{A}}\right) ^2 - k_{\scriptscriptstyle 0}^2 \varepsilon _{\mathcal{A}a}} \right] \cos \psi \sin \psi }{k_{\scriptscriptstyle 0}\alpha ^\pm _{\mathcal{A}} \left[ k_{\scriptscriptstyle 0}^2 \varepsilon _{\mathcal{A}a}\varepsilon _{\mathcal{A}c}-\left( \alpha ^\pm _{\mathcal{A}} \right) ^2 \varepsilon _{\mathcal{A}a}- \left( q^\pm \right) ^2 \left( \varepsilon _{\mathcal{A}a}\cos ^2 \psi + \varepsilon _{\mathcal{A}c}\sin ^2 \psi \right) \right] }} \\ \displaystyle {\pm \frac{1}{\eta _{\scriptscriptstyle 0}} \sqrt{\frac{\varepsilon _{\mathcal{A}a}-\varepsilon _{\mathcal{A}c}}{\varepsilon _{\mathcal{A}b}-\varepsilon _{\mathcal{A}a}}}} \\ \eta _{\scriptscriptstyle 0}^{-1} \end{array} \right] . \end{aligned}$$

The corresponding generalized eigenvector^32^ satisfying

16 $$\begin{aligned} \left( [\underline{\underline{P}}_{\mathcal{A}}]- \alpha ^\pm _{\mathcal{A}} [\underline{\underline{I}}]\right) {{^\cdot }}[\underline{w}^\pm _{\mathcal{A}} ]= [\underline{v}^\pm _{\mathcal{A}}]\end{aligned}$$

can be written as

17 $$\begin{aligned} {[\underline{w}^\pm _{\mathcal{A}}]} = \left[ \begin{array}{c} \displaystyle {\frac{A_6+A_7}{k_{\scriptscriptstyle 0}A_8 \left( \varepsilon _{\mathcal{A}a}-\varepsilon _{\mathcal{A}b}\right) } } \\ \displaystyle {\frac{k_{\scriptscriptstyle 0}^2 \left( \varepsilon _{\mathcal{A}a}-\varepsilon _{\mathcal{A}b}\right) A_8 +\left( A_6+A_7 \right) \left[ \left( q^\pm \right) ^2 \sin ^2 \psi - k_{\scriptscriptstyle 0}^2 \varepsilon _{\mathcal{A}b}\right] }{k_{\scriptscriptstyle 0}A_8 \left( q^\pm \right) ^2 \left( \varepsilon _{\mathcal{A}a}-\varepsilon _{\mathcal{A}b}\ \right) \sin \psi \cos \psi }} \\ \displaystyle {\frac{ \varepsilon _{\mathcal{A}a}\left( \alpha ^\pm _{\mathcal{A}} \right) ^2 \left( A_6+A_7 \right) -A_6 A_8}{\eta _{\scriptscriptstyle 0}\alpha ^\pm _{\mathcal{A}} A_8 \left( q^\pm \right) ^2 \left( \varepsilon _{\mathcal{A}a}-\varepsilon _{\mathcal{A}b}\ \right) \sin \psi \cos \psi } } \\ 0 \end{array} \right] , \end{aligned}$$

wherein $[\underline{\underline{I}}]$ is the $4\times 4$ identity matrix and the scalar quantities

18 $$\begin{aligned} \left. \begin{array}{l} A_6 = \displaystyle {\varepsilon _{\mathcal{A}a}\left[ \left( q^\pm \right) ^2 + \left( \alpha ^\pm _{\mathcal{A}} \right) ^2 - k_{\scriptscriptstyle 0}^2 \varepsilon _{\mathcal{A}a}\right] } \\ A_7 = \displaystyle {\left( q^\pm \right) ^2 \cos ^2 \psi \left[ \varepsilon _{\mathcal{A}a}-\varepsilon _{\mathcal{A}b}\pm \sqrt{\left( \varepsilon _{\mathcal{A}b}-\varepsilon _{\mathcal{A}a}\right) \left( \varepsilon _{\mathcal{A}a}- \varepsilon _{\mathcal{A}c}\right) }\tan \psi \right] + k_{\scriptscriptstyle 0}^2 \varepsilon _{\mathcal{A}c}\left( \varepsilon _{\mathcal{A}b}-\varepsilon _{\mathcal{A}a}\right) } \\ A_8= \displaystyle {\left( \alpha ^\pm _{\mathcal{A}} \right) ^2 \varepsilon _{\mathcal{A}a}- k_{\scriptscriptstyle 0}^2 \varepsilon _{\mathcal{A}b}\varepsilon _{\mathcal{A}c}+\left( q^\pm \right) ^2 \left( \varepsilon _{\mathcal{A}b}\cos ^2 \psi + \varepsilon _{\mathcal{A}c}\sin ^2 \psi \right) } \\ \end{array} \right\} . \end{aligned}$$

Hence, the general solution of Eq. (3)${}_1$ is expressed as

19 $$\begin{aligned}{}[\underline{f}(z)]= {\left( C_{\mathcal{A}1} [\underline{v}^\pm _{\mathcal{A}}]+ {k_{\scriptscriptstyle 0}} C_{\mathcal{A}2} \left\{ i z \, [\underline{v}^\pm _{\mathcal{A}} ]+ [\underline{w}^\pm _{\mathcal{A}}]\right\} \right) \exp \left( i \alpha ^\pm _{\mathcal{A}} z \right) },\qquad z > 0, \end{aligned}$$

for fields that decay as $z \rightarrow +\infty$. The complex-valued constants $C_{\mathcal{A}1}$ and $C_{\mathcal{A}2}$ herein are determined by the application of boundary conditions at $z=0$.

### Fields in material $\mathcal{B}$

The $4\times 4$ matrix $[\underline{\underline{P}}_{\mathcal{B}}]$ can be stated as^2^

20 $$\begin{aligned}{}[\underline{\underline{P}}_{\mathcal{B}}]= \left[ \begin{array}{cccc} 0&{}0&{} \displaystyle { \frac{\tau }{\omega \varepsilon _{\scriptscriptstyle 0}\varepsilon _{\mathcal{B}}}} &{} \displaystyle {\frac{k_{\scriptscriptstyle 0}^2 \varepsilon _{\mathcal{B}}- \nu _c }{\omega \varepsilon _{\scriptscriptstyle 0}\varepsilon _{\mathcal{B}}} } \\ 0&{}0&{} \displaystyle {\frac{ \nu _s -k_{\scriptscriptstyle 0}^2 \varepsilon _{\mathcal{B}} }{\omega \varepsilon _{\scriptscriptstyle 0}\varepsilon _{\mathcal{B}}} }&{} \displaystyle { -\frac{\tau }{\omega \varepsilon _{\scriptscriptstyle 0}\varepsilon _{\mathcal{B}}}} \\ \displaystyle { -\frac{\tau }{\omega \mu _{\scriptscriptstyle 0}}} &{} \displaystyle {\frac{ \nu _c -k_{\scriptscriptstyle 0}^2 \varepsilon _{\mathcal{B}}}{\omega \mu _{\scriptscriptstyle 0}} } &{}0&{}0 \\ \displaystyle {\frac{k_{\scriptscriptstyle 0}^2 \varepsilon _{\mathcal{B}}- \nu _s}{\omega \mu _{\scriptscriptstyle 0}} } &{} \displaystyle { \frac{\tau }{\omega \mu _{\scriptscriptstyle 0}}}&{}0&{}0 \end{array} \right] , \end{aligned}$$

and the *z*-directed components of the field phasors in material $\mathcal{B}$ are related to the *x*- and *y*-directed components as follows:

21 $$\begin{aligned} \left. \begin{array}{l} e_ z(z) = \displaystyle {\frac{q \left[ h_ x(z) \sin \psi - h_ y(z) \cos \psi \right] }{\omega \varepsilon _{\scriptscriptstyle 0}\varepsilon _{\mathcal{B}}}} \\ h_ z(z) = \displaystyle {\frac{q \left[ e_ y(z) \cos \psi - e_ x(z) \sin \psi \right] }{\omega \mu _{\scriptscriptstyle 0}}} \end{array} \right\} ,\qquad z < 0. \end{aligned}$$

The matrix $[\underline{\underline{P}}_{\mathcal{B}}]$ has two distinct eigenvalues: $\pm \alpha _{\mathcal{B}}$, with

22 $$\begin{aligned} \alpha _{\mathcal{B}} =- i \sqrt{q^2 - k_{\scriptscriptstyle 0}^2 \varepsilon _{\mathcal{B}}} . \end{aligned}$$

The sign of the square root in Eq. (22) must be such that $\text{ Im } \left\{ \alpha _{\mathcal{B}} \right\} < 0$, for surface-wave propagation. Each eigenvalue has algebraic multiplicity 2 and geometric multiplicity 2. The two linearly independent eigenvectors of $[\underline{\underline{P}}_{\mathcal{B}}]$ corresponding to the eigenvalue $\alpha _{\mathcal{B}}$ are given by

23 $$\begin{aligned} \left. \begin{array}{l} {[\underline{v}_{\mathcal{B}1}]} = \left[ \displaystyle {1 - \frac{ \nu _c}{k_{\scriptscriptstyle 0}^2 \varepsilon _{\mathcal{B}}}}, \quad \displaystyle {- \frac{ \tau }{k_{\scriptscriptstyle 0}^2 \varepsilon _{\mathcal{B}}}}, \quad 0, \quad \displaystyle {\frac{\alpha _{\mathcal{B}}}{\omega \mu _{\scriptscriptstyle 0}}} \right] ^T \\ {[\underline{v}_{\mathcal{B}2}]} = \left[ \displaystyle { \frac{\tau }{k_{\scriptscriptstyle 0}^2 \varepsilon _{\mathcal{B}}}}, \quad \displaystyle { \frac{ \nu _s}{k_{\scriptscriptstyle 0}^2 \varepsilon _{\mathcal{B}}} - 1}, \quad \displaystyle {\frac{\alpha _{\mathcal{B}}}{\omega \mu _{\scriptscriptstyle 0}}, \quad 0}\,\, \right] ^T \end{array} \right\} . \end{aligned}$$

Hence,

24 $$\begin{aligned}{}[\underline{f}(z)]= \left\{ C_{\mathcal{B}1} [\underline{v}_{\mathcal{B}1} ]+ C_{\mathcal{B}2} [\underline{v}_{\mathcal{B}2}]\right\} \exp \left( i \alpha _{\mathcal{B}} z \right) ,\qquad z < 0, \end{aligned}$$

is the general solution of Eq. (3)${}_2$ for surface waves that decay as $z \rightarrow -\infty$, wherein the complex-valued constants $C_{\mathcal{B}1}$ and $C_{\mathcal{B}2}$ have to be determined by applying boundary conditions at $z=0$.

### Boundary conditions at the interface $z=0$

Across the interface plane $z=0$, the tangential components of the electric and magnetic field phasors must be continuous. Accordingly, the applicable four boundary conditions are compactly expressed as

25 $$\begin{aligned}{}[\underline{f}(0^+)]= [\underline{f}(0^-)]. \end{aligned}$$

The combination of Eqs. (11) and (24), along with Eq. (25), yields

26 $$\begin{aligned} {[\underline{\underline{Y}}]} {{^\cdot }}\left[ \, C_{\mathcal{A}1}, \quad C_{\mathcal{A}2}, \quad C_{\mathcal{B}1}, \quad C_{\mathcal{B}2} \, \right] ^T = \left[ \, 0, \quad 0, \quad 0, \quad 0 \, \right] ^T. \end{aligned}$$

The $4\times 4$ characteristic matrix $[\underline{\underline{Y}}]$ must be singular for surface-wave propagation^2^; hence, the dispersion equation

27 $$\begin{aligned} \left| {[\underline{\underline{Y}}]} \right| = 0 \end{aligned}$$

emerges. The explicit representation of Eq. (27) is too unwieldy for reproduction here, but *q* can be numerically extracted from Eq. (27), by the Newton–Raphson method^33^ for example.

If $\psi$ is replaced by $-\psi$ or by $\pi \pm \psi$ then the dispersion equation (27) is unchanged. Accordingly, in the following numerical investigation of Dyakonov and Dyakonov–Voigt surface waves, attention is restricted to the quadrant $0 \le \psi \le \pi /2$.

## Numerical studies

Let us now present and discuss numerical solutions to the dispersion equation (27). Numerical values were chosen for the relative permittivity parameters for this purpose, keeping in mind that the inequality (14) must be satisfied for material $\mathcal{A}$ in order for Dyakonov–Voigt surface waves to exist. Candidates materials in the following two categories to function as material $\mathcal{A}$ were explored:

(i) naturally occurring minerals with relatively modest birefringence and
(ii) engineered materials with large birefringence.

### Naturally occurring material $\mathcal{A}$

Three biaxial minerals were selected to function as material $\mathcal{A}$ for the first numerical study: crocoite, tellurite, and cerussite. The relative permittivity scalars of these three minerals, along with the values of their biaxiality $\beta _{\mathcal{A}}=\cos (2\delta _{\mathcal{A}})$ where $\delta _{\mathcal{A}} =$ $\cos ^{-1}$ $\sqrt{\left( \varepsilon _{\mathcal{A}b}-\varepsilon _{\mathcal{A}a}\right) /\left( \varepsilon _{\mathcal{A}b}- \varepsilon _{\mathcal{A}c}\right) }$ and birefringence $\Delta n_{\mathcal{A}} = \sqrt{\varepsilon _{\mathcal{A}b}} - \sqrt{\varepsilon _{\mathcal{A}c}}$, are provided in Table 1. Both optic ray axes of these minerals are arranged to lie in the *xy* plane with the *y* axis as the bisector, the angle $\delta _{\mathcal{A}}$ being the half-angle between the two optic ray axes. Whereas crocoite has moderately positive biaxiality, tellurite has very weakly positive biaxiality, and cerussite has strongly negative biaxiality^34^. While all three minerals were taken to be orthorhombic^28^, we note parenthetically that crocoite also exists in monoclinic form^35^.

**Table 1:** Relative permittivity scalars and derivative constitutive parameters of three minerals.

Mineral	$\varepsilon _{\mathcal{A}a}$	$\varepsilon _{\mathcal{A}b}$	$\varepsilon _{\mathcal{A}c}$	$\delta _{\mathcal{A}}$	$\beta _{\mathcal{A}}$	$\Delta n_{\mathcal{A}}$
Crocoite	5.6169	7.0756	5.3361	$23.69^\circ$	0.677	0.350
Tellurite	4.7524	5.5225	4.0000	$44.67^\circ$	0.012	0.350
Cerussite	4.3015	4.3098	3.2508	$84.92^\circ$	$-0.984$	0.273

**Figure 2:**
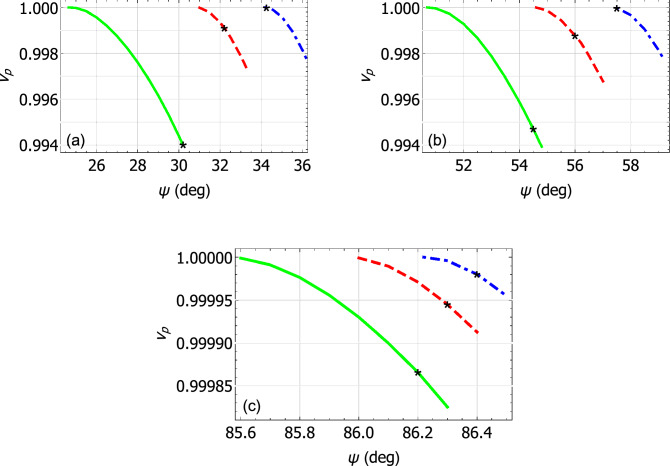
Relative phase speed $v_p$ plotted versus $\psi$ for Dyakonov surface waves. (**a**) Material $\mathcal{A}$ is crocoite and $\varepsilon _{\mathcal{B}}=5.622$ (green solid curve), 5.732 (red dashed curve), or 5.810 (blue broken-dashed curve). (**b**) Material $\mathcal{A}$ is tellurite and $\varepsilon _{\mathcal{B}}=4.843$ (green solid curve), 4.931 (red dashed curve), or 4.999 (blue broken-dashed curve). (**c**) Material $\mathcal{A}$ is cerussite and $\varepsilon _{\mathcal{B}}=4.3025$ (green solid curve), 4.3035 (red dashed curve), or 4.3042 (blue broken-dashed curve). Every Dyakonov–Voigt surface wave is identified by a black star on each curve.

Values of the relative phase speed

28 $$\begin{aligned} v_p = \frac{k_{\scriptscriptstyle 0}\sqrt{\varepsilon _{\mathcal{B}}}}{q} \end{aligned}$$

of Dyakonov surface waves are plotted versus the propagation angle $\psi \in (0,\pi /2)$ in Fig. 2a–c. For these calculations, (a) $\varepsilon _{\mathcal{B}} \in \left\{ 5.622, 5.732, 5.810 \right\}$ when material $\mathcal{A}$ is crocoite; (b) $\varepsilon _{\mathcal{B}}\in \left\{ 4.843, 4.931, 4.999 \right\}$ when material $\mathcal{A}$ is tellurite; and (c) $\varepsilon _{\mathcal{B}}\in \left\{ 4.3025, 4.3035, 4.3042 \right\}$ when material $\mathcal{A}$ is cerussite. For all three minerals considered as material $\mathcal{A}$, the relative phase speed decreases as $\psi$ increases; also, the angular existence domain (i.e., the range of $\psi$) for Dyakonov surface-wave propagation narrows as the value of $\varepsilon _{\mathcal{B}}$ increases.

A solitary point on each curve in Fig. 2, denoted by a black star, indicates the existence of a Dyakonov–Voigt surface wave with surface wavenumber $q = q^-$. The alternative value of *q*, namely $q = q^+$, does not deliver a surface wave. Each Dyakonov–Voigt surface wave corresponds to an exceptional point of the matrix $[\underline{\underline{P}}_{\mathcal{A}}]$. For each mineral, the propagation angle for each Dyakonov–Voigt surface wave gets closer to the lower bound of the angular existence domain for Dyakonov surface waves as $\varepsilon _{\mathcal{B}}$ increases.

**Figure 3:**
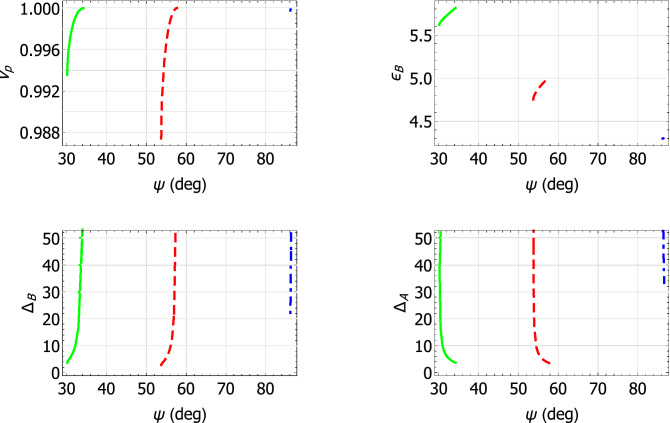
Plots of $v_p$ , $\varepsilon _{\mathcal{B}}$, $\Delta _{\mathcal{A}}$, and $\Delta _{\mathcal{B}}$ versus $\psi$ for Dyakonov–Voigt surface waves. Material $\mathcal{A}$ is crocoite (green solid curves), tellurite (red dashed curve), and cerussite (blue broken-dashed curve).

The characteristics of the Dyakonov–Voigt surface waves identified in Fig. 2 are further delineated in Fig. 3 wherein $v_p$, $\varepsilon _{\mathcal{B}}$, and the penetration depths in materials $\mathcal{A}$ and $\mathcal{B}$, as given by

29 $$\begin{aligned} \left. \begin{array}{l} \Delta _{\mathcal{A}} = \displaystyle {\frac{k_{\scriptscriptstyle 0}}{\text{ Im } \left\{ \alpha _{\mathcal{A}} \right\} }} \\ \Delta _{\mathcal{B}} = \displaystyle {\frac{k_{\scriptscriptstyle 0}}{- \text{ Im } \left\{ \alpha _{\mathcal{B}} \right\} }} \end{array} \right\} , \end{aligned}$$

respectively, are plotted against $\psi$. For all three minerals considered, the quantities $v_p$, $\varepsilon _{\mathcal{B}}$, and $\Delta _{\mathcal{B}}$ increase monotonically whereas $\Delta _{\mathcal{A}}$ decreases monotonically, as $\psi$ increases. For each mineral, the range of $\psi$ for Dyakonov–Voigt surface waves in Fig. 3 is bounded below by the value of $\psi$ for which $\text{ Im } \left\{ \alpha _{\mathcal{A}} \right\} = 0$ and bounded above by the value of $\psi$ for which $\text{ Im } \left\{ \alpha _{\mathcal{B}} \right\} = 0$. As $\psi$ decreases towards its lower bound, the corresponding value of $\varepsilon _{\mathcal{B}}$ approaches $\varepsilon _{\mathcal{A}a}$.

**Figure 4:**
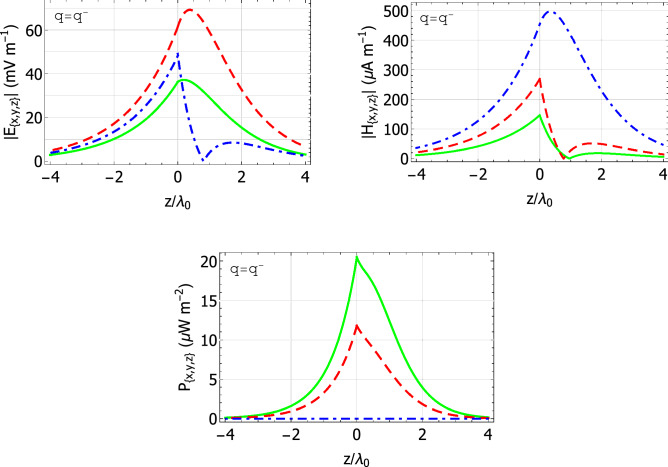
Spatial field profiles for a Dyakonov–Voigt surface wave: components of the quantities $|\underline{E} (z\hat{\underline{u}}_{\,z}) {{^\cdot }}\underline{n}|$, $|\underline{H} (z\hat{\underline{u}}_{\,z}) {{^\cdot }}\underline{n}|$, and $\underline{P} (z\hat{\underline{u}}_{\,z}) {{^\cdot }}\underline{n}$ are plotted versus $z/\lambda _{\scriptscriptstyle 0}$. Material $\mathcal{A}$ is crocoite, $\varepsilon _{\mathcal{B}}=5.732$, ${q=q^-=2.39639k_{\scriptscriptstyle 0}}$, $\psi =32.2 ^\circ$, and $C_{\mathcal {B}3} = 1$ V m${}^{-1}$. Key: $\underline{n} = \hat{\underline{u}}_x$ green solid curves; $\underline{n} = \hat{\underline{u}}_y$ red dashed curves; $\underline{n} = \hat{\underline{u}}_z$ blue broken-dashed curves.

The localization of the Dyakonov–Voigt surface when $\mathcal{A}$ is crocoite and $\varepsilon _{\mathcal{B}} = 5.732$ ($\psi = 32.2^\circ$ in Fig. 2a) can be observed in Fig. 4. Therein, the quantities $|\underline{E} (z\hat{\underline{u}}_{\,z}) {{^\bullet }}\underline{n}|$ and $|\underline{H} (z\hat{\underline{u}}_{\,z}) {{^\bullet }}\underline{n}|$ are plotted versus $z/\lambda _{\scriptscriptstyle 0}$ for $\underline{n} \in \left\{ \hat{\underline{u}}_x, \hat{\underline{u}}_y, \hat{\underline{u}}_z\right\}$ and $C_{\mathcal {B}3} = 1$ V m${}^{-1}$. Also plotted in Fig. 4 are the quantities $\underline{P} (z\hat{\underline{u}}_{\,z}) {{^\bullet }}\underline{n}$ for $\underline{n} \in \left\{ \hat{\underline{u}}_x, \hat{\underline{u}}_y, \hat{\underline{u}}_z\right\}$, with

30 $$\begin{aligned} \underline{P} (\underline{r}) = \frac{1}{2} \text{ Re } \left[ \, \underline{E} (\underline{r}) \times \underline{H}^* (\underline{r}) \, \right] \end{aligned}$$

being the time-averaged Poynting vector. The Dyakonov–Voigt surface wave in Fig. 4 is rather more tightly localized to the plane $z=0$ in material $\mathcal{B}$ than in material $\mathcal{A}$; even so, in both half-spaces the surface wave is essentially contained within $|z| < 4 \lambda _{\scriptscriptstyle 0}$. Also, there is no energy flow directed normally to the interface plane in either half-space.

Generally, the spatial field profiles for the other eight Dyakonov–Voigt surface waves identified in Fig. 2 are similar to those presented in Fig. 4. Examination of those field profiles revealed the following trend: if the propagation angle of the Dyakonov–Voigt surface wave is closer to the upper bound of the angular existence domain of Dyakonov surface waves then the Dyakonov–Voigt surface wave is less tightly bound in the half-space $z>0$ to the interface $z=0$, whereas if the propagation angle of the Dyakonov–Voigt surface wave is closer to the lower bound of the angular existence domain of Dyakonov surface waves then the Dyakonov–Voigt surface wave is less tightly bound in the half-space $z<0$ to the interface $z=0$.

### Engineered material $\mathcal{A}$

Now we turn to scenarios in which material $\mathcal{A}$ is an engineered material with high birefringence. Such engineered materials may be conceptualized as homogenized composite materials^36^, for example, arising from component materials made up from elongated particles or fibers. By judiciously embedding highly elongated component particles or fibers in a host material, homogenized composite materials that exhibit exceedingly high degrees of anisotropy may be realized^37^. The relative permittivity scalars and derivative constitutive parameters of three chosen engineered materials labeled I, II, and III are provided in Table 2, all with strongly positive biaxiality.

**Table 2:** Relative permittivity scalars and derivative constitutive parameters of three engineered materials.

Engineered material	$\varepsilon _{\mathcal{A}a}$	$\varepsilon _{\mathcal{A}b}$	$\varepsilon _{\mathcal{A}c}$	$\delta _{\mathcal{A}}$	$\beta _{\mathcal{A}}$	$\Delta n_{\mathcal{A}}$
I	2.0	14.0	1.9	$5.22^\circ$	0.983	2.327
II	2.0	9.0	1.99	$2.16^\circ$	0.997	1.586
III	2.0	6.5	2.0	$0.085^\circ$	1.0	1.135

**Figure 5:**
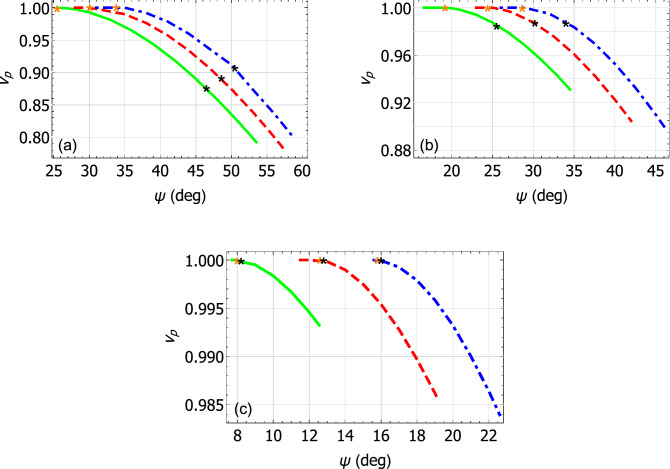
Relative phase speed $v_p$ plotted versus $\psi$ for Dyakonov surface waves. (**a**) Material $\mathcal{A}$ is engineered material I and $\varepsilon _{\mathcal{B}}=2.7$ (green solid curve), 3 (red dashed curve), or 3.3 (blue broken-dashed curve). (**b**) Material $\mathcal{A}$ is engineered material II and $\varepsilon _{\mathcal{B}}=2.3$ (green solid curve), 2.5 (red dashed curve), or 2.7 (blue broken-dashed curve). (**c**) Material $\mathcal{A}$ is engineered material III and $\varepsilon _{\mathcal{B}}=2.04$ (green solid curve), 2.1 (red dashed curve), or 2.16 (blue broken-dashed curve). On each curve, a Dyakonov–Voigt surface wave with $q=q^-$ is identified by a black star and with $q=q^+$ is identified by an orange star.

Values of the relative phase speed $v_p$ of Dyakonov surface waves are plotted versus the propagation angle $\psi \in (0,\pi /2)$ in Fig. 5a–c. For these calculations, (a) $\varepsilon _{\mathcal{B}} \in \left\{ 2.7, 3, 3.3 \right\}$ when material $\mathcal{A}$ is engineered material I; (b) $\varepsilon _{\mathcal{B}}\in \left\{ 2.3, 2.5, 2.7 \right\}$ when material $\mathcal{A}$ is engineered material II; and (c) $\varepsilon _{\mathcal{B}}\in \left\{ 2.04, 2.1, 2.16 \right\}$ when material $\mathcal{A}$ is engineered material III. For all three engineered materials considered, $v_p$ decreases as $\psi$ increases. The angular existence domain for Dyakonov surface waves widens in Fig. 5 as the value of $\varepsilon _{\mathcal{B}}$ increases, in contrast to the trend observable in Fig. 2 when material $\mathcal{A}$ has a small birefringence. Also, the angular existence domains for Dyakonov surface waves in Fig. 5 for the three engineered materials serving as material $\mathcal{A}$ are substantially larger than those in Fig. 2 for the three minerals in the same role.

On each curve in Fig. 5 there are two stars. These represent the coexistence of *two* Dyakonov–Voigt surface waves: the black stars denote $q = q^-$ and the orange stars denote $q = q^+$. Each pair of Dyakonov–Voigt surface waves corresponds to a pair of exceptional points of the matrix $[\underline{\underline{P}}_{\mathcal{A}}]$. The separation between the propagation angles for $q = q^-$ and $q = q^+$ is the greatest for the engineered material with the largest difference between $\varepsilon _{\mathcal{A}a}$ and $\varepsilon _{\mathcal{A}c}$.

**Figure 6:**
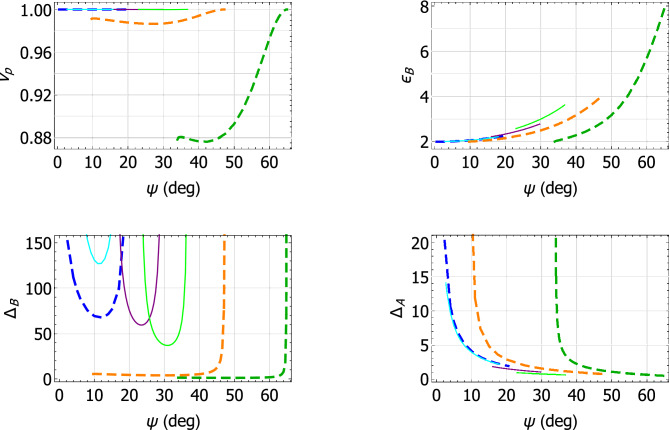
Plots of $v_p$ , $\varepsilon _{\mathcal{B}}$, $\Delta _{\mathcal{A}}$, and $\Delta _{\mathcal{B}}$ versus $\psi$ for Dyakonov–Voigt surface waves. Material $\mathcal{A}$ is: engineered material I with $q=q^+$ (green solid curves) and $q=q^-$ (dark green dashed curves); engineered material II with $q=q^+$ (purple solid curves) and $q=q^-$ (orange dashed curves); and engineered material III with $q=q^+$ (cyan solid curves) and $q=q^-$ (blue dashed curves).

Further insights into the characteristics of the Dyakonov–Voigt surface waves identified in Fig. 5 are gleaned from Fig. 6, wherein $v_p$, $\varepsilon _{\mathcal{B}}$, $\Delta _{\mathcal{A}}$, and $\Delta _{\mathcal{B}}$ are plotted against $\psi$. For all three engineered materials considered, $\Delta _{\mathcal{A}}$ decreases monotonically and $\varepsilon _{\mathcal{B}}$ increases monotonically as $\psi$ increases. For $q = q^+$, we have $\text{ Im } \left\{ \alpha _{\mathcal{B}} \right\} = 0$ when $\psi$ takes its smallest value and its largest value. The range of $\psi$ for Dyakonov–Voigt surface waves in Fig. 6 for $q = q^-$ is bounded above by the value of $\psi$ for which $\text{ Im } \left\{ \alpha _{\mathcal{B}} \right\} = 0$. As $\psi$ decreases toward its lower bound, the corresponding value of $\varepsilon _{\mathcal{B}}$ for $q = q^-$ approaches $\varepsilon _{\mathcal{A}a}$.

**Figure 7:**
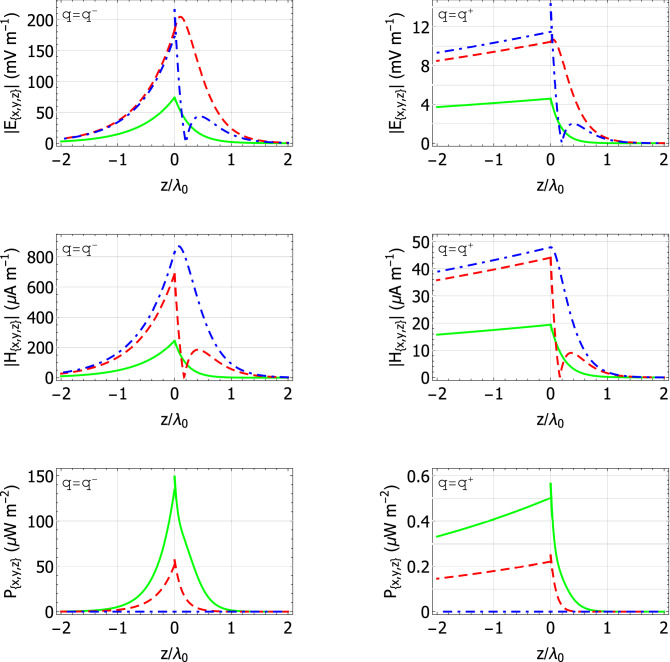
Spatial field profiles for two Dyakonov–Voigt surface waves: components of the quantities $|\underline{E} (z\hat{\underline{u}}_{\,z}) {{^\cdot }}\underline{n}|$, $|\underline{H} (z\hat{\underline{u}}_{\,z}) {{^\cdot }}\underline{n}|$, and $\underline{P} (z\hat{\underline{u}}_{\,z}) {{^\cdot }}\underline{n}$ are plotted versus $z/\lambda _{\scriptscriptstyle 0}$. Material $\mathcal{A}$ is engineered material II, $\varepsilon _{\mathcal{B}}=2.5$, and $C_{\mathcal {B}3} = 1$ V m${}^{-1}$. Left: $q = q^-={1.602k_{\scriptscriptstyle 0}}$ and $\psi =30.1847^\circ$. Right: $q = q^+={1.58123k_{\scriptscriptstyle 0}}$ and $\psi =24.4068^\circ$. Key: $\underline{n} = \hat{\underline{u}}_x$ green solid curves; $\underline{n} = \hat{\underline{u}}_y$ red dashed curves; $\underline{n} = \hat{\underline{u}}_z$ blue broken-dashed curves.

Lastly, we turn to the localization of the pairs of Dyakonov–Voigt surface waves identified in Fig. 5. The spatial field profiles when material $\mathcal{A}$ is engineered material II and $\varepsilon _{\mathcal{B}} = 2.5$ are provided in Fig. 7 with $C_{\mathcal {B}3} = 1\,\hbox {V m}{}^{-1}$. The corresponding propagation angles for the Dyakonov–Voigt surface waves with $q=q^-$ and $q^+$ are $\psi = 30.1847^\circ$ and $24.4068^\circ$, respectively.

The spatial profiles for the Dyakonov–Voigt surface wave for $q=q^-$ in Fig. 7 resemble those provided in Fig. 4, except that the latter profiles are more symmetric about the interface $z=0$ than the former. Examination of the spatial profiles for all nine Dyakonov–Voigt surface waves for $q=q^-$ identified in Fig. 5 revealed the following trend: if the propagation angle of the Dyakonov–Voigt surface wave is closer to the upper bound of the angular existence domain of Dyakonov surface waves then the Dyakonov–Voigt surface wave is less tightly bound in the half-space $z>0$ to the interface $z=0$, whereas if the propagation angle of the Dyakonov–Voigt surface wave is closer to the lower bound of the angular existence domain of Dyakonov surface waves then the Dyakonov–Voigt surface wave is less tightly bound in the half-space $z<0$ to the interface $z=0$.

The spatial profiles for the Dyakonov–Voigt surface wave for $q=q^+$ in Fig. 7 are substantially different from those for the Dyakonov–Voigt surface wave for $q=q^-$ in the same figure: the surface wave for $q=q^+$ is much less tightly localized to the interface in the half-space $z<0$ and slightly more tightly localized to the interface in the half-space $z>0$. In addition, the magnitudes of the energy flows in the *x* and *y* directions associated with the surface wave for $q=q^+$ are much less than the corresponding energy flows for the surface wave for $q=q^-$.

The spatial field profiles for the other eight Dyakonov–Voigt surface waves identified in Fig. 5 for $q=q^+$ are qualitatively similar to those presented in Fig. 7. Examination of the spatial profiles for all nine Dyakonov–Voigt surface waves for $q=q^+$ identified in Fig. 5 revealed that, as $\varepsilon _{\mathcal{B}}$ increases, the Dyakonov–Voigt surface wave becomes more tightly bound to the interface in the half-space $z>0$ for fixed values of $\varepsilon _{\mathcal{A}a}$, $\varepsilon _{\mathcal{A}b}$, and $\varepsilon _{\mathcal{A}c}$.

## Closing remarks

Our theoretical and numerical investigations have revealed that the planar interface of an orthorhombic dielectric material and an isotropic dielectric material can guide two Dyakonov–Voigt surface waves in each quadrant of the interface plane, provided that the birefringence of the orthorhombic partnering material is sufficiently large. The two Dyakonov–Voigt surface waves propagate at different phase speeds, in different directions, and with different penetration depths. Also, the Dyakonov–Voigt surface wave with the lower relative phase speed is much more tightly bound to the interface in the isotropic dielectric partnering material. In contrast, the planar interface of a uniaxial dielectric material and an isotropic dielectric material can guide only one Dyakonov–Voigt surface wave in each quadrant of the interface plane.

The canonical boundary-value problem investigated herein has yielded insights into the fundamental characteristics of Dyakonov–Voigt surface-wave propagation. Issues concerning the excitation of these exceptional surface waves in experimental configurations, such as the prism-coupled configuration^2^, shall be pursued in future studies. The numerical results presented in “[Sec Sec10]” are based on naturally occurring partnering materials while the numerical results in “[Sec Sec11]” are based on realistic engineered partnering materials. Accordingly, in principle, the theoretical analysis presented in “Canonical boundary-value problem” and numerical results presented in “Numerical studies” may be readily confirmed by experimental means.

Lastly, let us emphasize that the coexistence of two Dyakonov–Voigt surface waves guided by the planar interface of a biaxial dielectric material and an isotropic dielectric material is wholly independent of the coexistence of two different plane waves, both extraordinary^26^ and with different phase speeds, that can propagate inside a biaxial dielectric material^26,27^. Plane waves that propagate in the bulk of a homogeneous material are entirely different from surface waves guided by the planar interface of two dissimilar homogeneous materials.
